# Jean-Martin Charcot: the polymath

**DOI:** 10.1055/s-0043-1775984

**Published:** 2023-10-29

**Authors:** Carlos Henrique Ferreira Camargo, Léo Coutinho, Ylmar Correa Neto, Eliasz Engelhardt, Pericles Maranhão Filho, Olivier Walusinski, Hélio Afonso Ghizoni Teive

**Affiliations:** 1 Universidade Federal do Paraná, Programa de Pós-Graduação em Medicina Interna, Disciplina de Doenças Neurodegenerativas, Curitiba PR, Brazil.; 2 Universidade Federal de Santa Catarina, Departamento de Medicina Interna, Serviço de Neurologia, Florianópolis SC, Brazil.; 3 Universidade Federal do Rio de Janeiro, Instituto de Neurologia Deolindo Couto e Instituto de Psiquiatria, Rio de Janeiro RJ, Brazil.; 4 Universidade Federal do Rio de Janeiro, Departamento de Clínica Médica, Serviço de Neurologia, Rio de Janeiro RJ, Brazil.; 5 Clínica Privada, Brou, France.; 6 Universidade Federal do Paraná, Departamento de Clínica Médica, Serviço de Neurologia, Curitiba PR, Brazil.

**Keywords:** History, Neurology, Neuropathology, Internal Medicine, Psychiatry, Psychology, Polymath, História, Neurologia, Neuropatologia, Medicina Interna, Psiquiatria, Psicologia, Polímata

## Abstract

Jean-Martin Charcot, widely regarded as a leading founder of modern neurology, made substantial contributions to the understanding and characterization of numerous medical conditions. His initial focus was on internal medicine, later expanding to include neuropathology, general neurology, and eventually emerging fields such as neuropsychology and neuropsychiatry. Furthermore, Charcot's intellectual pursuits extended beyond medicine, encompassing research in art history, medical iconography, sociology, religious studies, and the arts, solidifying his status as a polymath.

## Resumo

Jean-Martin Charcot, amplamente considerado como um proeminente fundador da neurologia moderna, fez contribuições substanciais para a compreensão e a caracterização de várias condições médicas. Seu foco inicial era a medicina interna, expandindo-se posteriormente para incluir a neuropatologia, a neurologia geral e, por fim, campos emergentes como a neuropsicologia e a neuropsiquiatria. Além disso, as buscas intelectuais de Charcot foram além da medicina, abrangendo pesquisas em história da arte, iconografia médica, sociologia, estudos religiosos e artes, solidificando seu status de polímata.

## INTRODUCTION

In 2020, Peter Burke, Professor Emeritus of Cultural History at the University of Cambridge, published the book entitled “The Polymath. A cultural history from Leonardo da Vinci to Susan Sontag.” 1 In the introduction to this famous book, the author defined a polymath as “someone who is interested in many subjects and learns many subjects.” Five hundred Western polymaths were presented, including preeminent and controversial physicians such as Erasmus Darwin (1731–1802), Paul Broca (1824–1880), Sigmund Freud (1856–1939) and Oliver Sacks (1933–2015), but interesting omits Jean-Martin Charcot (1825–1893). 1

Apart from being widely considered a leading founder of modern neurology, Charcot's revolutionary ideas about neurological diseases continue to shape the practices of clinicians and scientists to this very day. Furthermore, Charcot's brilliance extended well beyond the realm of neurology, as he was actively engaged in diverse areas of medicine, psychology, arts, politics, diplomacy, religion, and philosophy. 2 3 4 5 This breadth of knowledge and involvement undoubtedly qualifies him as a polymath. 1

The purpose of this article is to briefly present Charcot's main contributions to neurology and beyond.

## CHARCOT: A SHORT BIOGRAPHY

**Figure 1:**
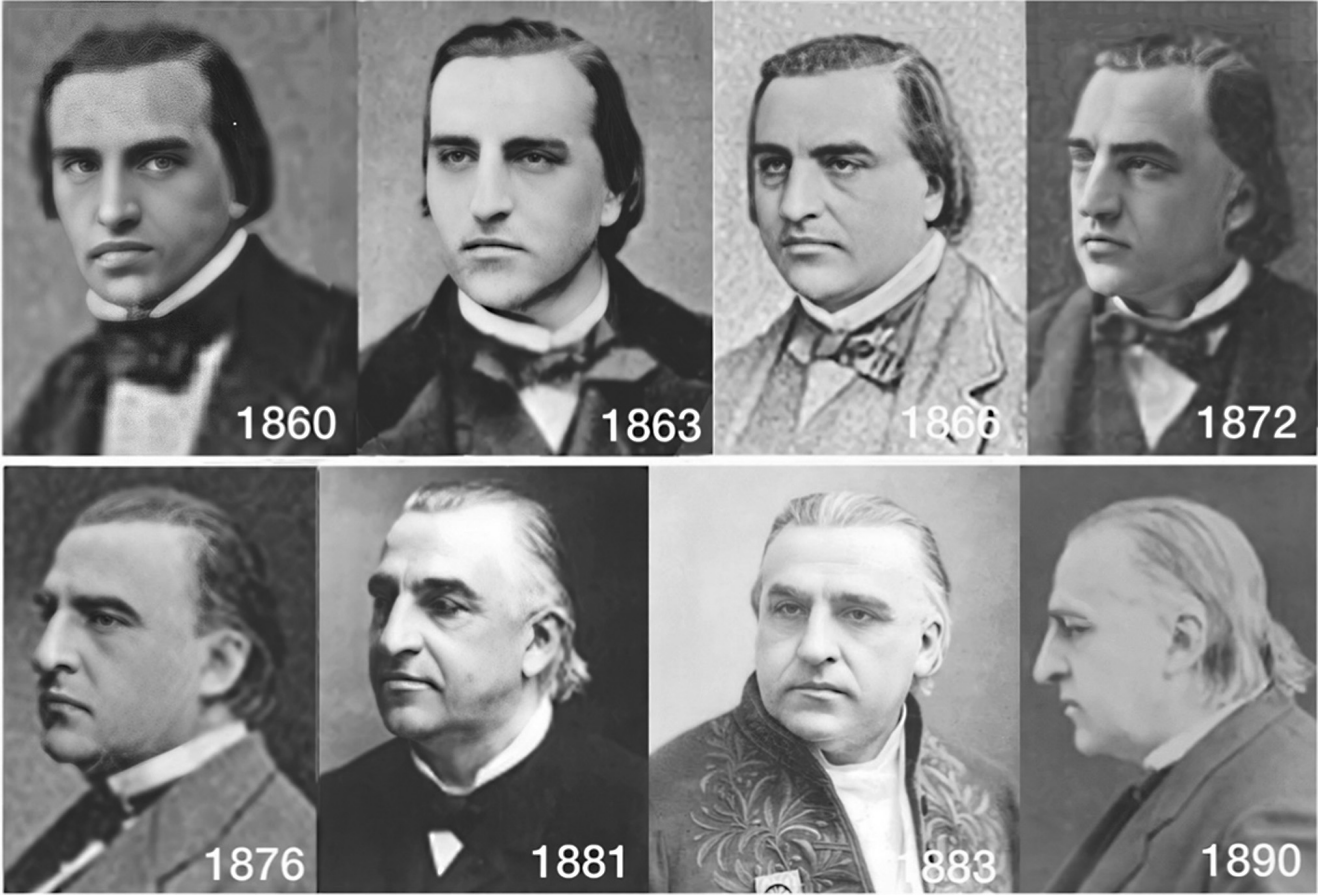
Jean-Martin Charcot (1825–1893). Evolution of Charcot's appearance from his admission to *La Salpêtrière* (1860) to three years before his death (1890). Public domain ( https://commons.wikimedia.org : 1863, 1872, 1881, 1883) and personal collection (Walusinki: 1860, 1866, 1876, 1890).

Jean-Martin Charcot ( Figure 1 ) was born on November 29, 1825, at 1 rue du Faubourg Poissonnière, 9th arrondissement, Paris, France. His parents were Simon-Pierre Charcot (1798–1863) and Jeanne-Georgette Saussier (1808–1839). He was the eldest of four siblings and was raised in a lower-middle-class artisan family. 2 3 6 7 8 9 Charcot's family background was deeply connected to the manufacture and decoration of carriages, with his father, maternal grandfather, and one of his brothers involved in this trade. 2 3 6 7 8

During his youth, Charcot was described as a slender individual with long black hair combed back, and he tended to keep to himself, rarely interacting with his peers. 8 At the age of 38, in 1864, Charcot married Augustine-Victoire Laurent (1834–1899), a widow with a 10-year-old daughter named Maria Charlotte Thérèse Durvis (1854–1936). Together, Charcot and Augustine-Victoire had two children: Jeanne Marie Amélie Claudine Charcot (1865–1940) and Jean-Baptiste Charcot (1867–1937). 2 6 7

Charcot pursued his education and graduated in letters before enrolling in the *École de Médecine* in Paris in 1843. After successfully passing the external competition in December 1845, Charcot was appointed provisional intern in 1847, during which time he earned the admiration and confidence of his mentor, Pierre Rayer (1793–1867). 2 10 Upon Rayer's recommendation, Charcot was appointed as an *agrégé* (associate professor) at the *École de Médecine* and introduced to the *Societé de Biologie* in 1851, which provided him with valuable access to the scientific community. 8

Between 1853 and 1855, Charcot served as a *chef de clinique* at the *Hôpital de la Charité* under the leadership of his master Pierre Adolphe Piorry (1794–1879). 8 In May 1856, he was appointed to the Bureau Central of Paris ( *médicin des hôpitaux* ). 10 In 1857, due to difficulties in expressing himself orally and presenting a controversial thesis on “ *De l'expectation en médicine* ,” which was deemed insufficient, he failed in his attempt to become an *agrégé* . Nevertheless, he succeeded in his second attempt in 1860 and assumed the position of *agrégé* intern of medicine and legal medicine, later being promoted to *agrégé* in exercise. 8 In 1862, Charcot became the *chef de service* de *La Salpêtrière* , and in 1872, he won a public competition for the *Chaire d'Anatomie Pathologique* . 2 3 6 7 In 1882, he became a clinical professor at the *École de Médecine* of the University of Paris and assumed the position of *Chaire de Clinique des Maladies du Système Nerveux* , a role he excelled in until his passing on August 16, 1893. 2 3 6 7

**Figure 2:**
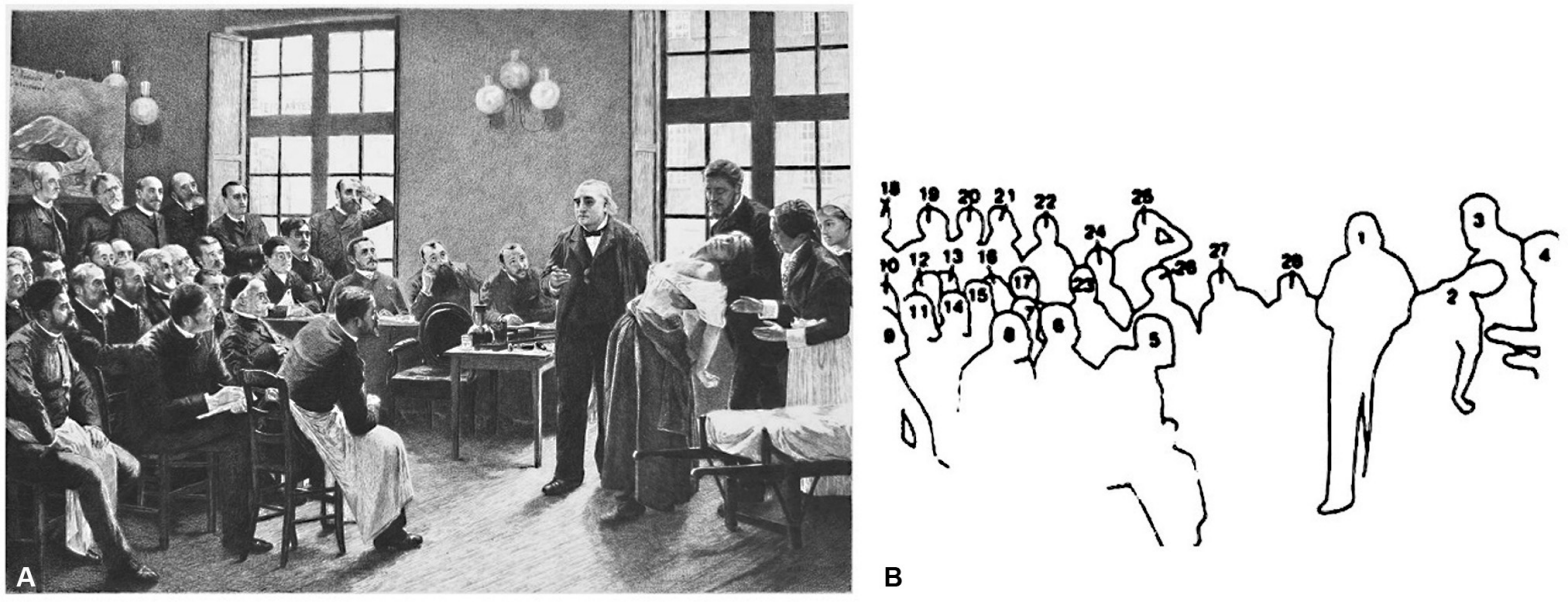
*Une Leçon Clinique à La Salpêtriére* , from 1887, by André Brouillet. ( **A** ). A engraved reproduction of Brouillet's painting by Henri Dochy (1851–1915). (Corrêa Neto, personal collection). ( **B** ). Charcot and other characters of the painting in a drawing with the numbered silhouettes: 1-JM Charcot; 2- Blanche Wittman, the patient; 3- J Babinski; 4- Mlle. Bottard, head nurse; 5- GG de la Tourette; 6- R Vigouroux; 7- H Parinaud; 8- H Berbez; 9- A Londe; 10- G Guinon; 11- L le Bas; 12- A Gombaut; 13- A Arène; 14- J Claretie; 15- A Naquet; 16- Bourneville; 17- G Bellet; 18- V Cornil; 19- P Burty; 20- M Debove; 21- M Durval; 22- JB Charcot; 23- P Berbez; 24- E Brissaud; 25- A Joffroy; 26- P Marie; 27- CS Féré; 28- P Richer.

During his tenure, the neurological school at *La Salpêtrière* gained global recognition as the “Mecca of Neurology.” 2 3 6 7 Charcot's group of pupils included renowned names like Joseph Babiński (1857–1932), and other disciples who also worked in his private practice as secretaries, including Désiré Magloire Bourneville (1840–1909), Charles Féré (1852–1907), Pierre Marie (1853–1940), Paul Richer (1849–1933), Georges Gilles de la Tourette (1857–1904), and Édouard Brissaud (1852–1909). 2 3 11 12 13 14 Additionally, 33 interns were part of Charcot's group at *La Salpêtrière* between 1862 and 1893, some of whom gained significant prominence in the field of neurology. 11 Many of them are in the world-renowned painting *Une Leçon Clinique à La Salpêtriére* , from 1887, by André Brouillet (1857–1914) ( Figure 2 ). 12 Internationally acclaimed neurologists sought internships at *La Salpêtrière* under Charcot's guidance, including James Jackson Putnam (1846–1918), M. Allen Starr (1854–1932), William James (1842–1910), Bernard Sachs (1858–1944), Vladimir M. Bechterew (1857–1927), Liverij O. Darkshevitch (1858–1925), Aleksej Y. Kozhevnikov (1836–1902), Gheorghe Marinescu (1863–1938), and Sigmund Freud. 2 3 4 6 15

A noteworthy fact that indirectly underscores Charcot's importance in the Parisian scientific community is his participation as a member of the thesis jury of the *École de Médecine* in Paris. 16 In the period between 1862 and 1893, a total of 12,500 theses were submitted to the *École de Médecine,* out of which 3,663 were in the field of neuropsychiatry. 17 Charcot, along with Alfred Vulpian (1826–1887) and Alexandre Axenfeld (1825–1876), were involved in 1,774 of these theses, with Charcot personally participating in 603 of them. 16

The intense scientific production of Charcot, as well as that of his group of disciples at the *La Salpêtrière* solidified him as the first professor of diseases of the nervous system in the world, as well as the most emblematic neurologist of the 19 ^th^ century. 3 4 15 17 18 19 20 Despite not being known for his eloquence, Charcot's lectures were characterized by exceptional clarity and the visual impact of his illustrations. He skillfully engaged his audience, occasionally adding dramatic flair when presenting various classic neurological syndromes. This captivating teaching style was evident both in his *Leçons du Mardi* (Tuesday lessons), and the scientific meetings held on Thursdays. 2 3 4 15 17 18 19 20

His international fame brought several personalities from Europe, and around the world people to Paris for neurological medical consultations, including the Grand Duke of Russia Vladimir Alexandrovich (1847–1909), the Brazilian Emperor Pedro II (1825–1891), Cardinal Charles Lavigerie (1825–1892), among other political leaders, artists, writers, and philosophers. 2 3 4 5 6 7 21 Furthermore, many of these distinguished personalities frequented Charcot's residence at 217 Boulevard Saint-Germain, especially during winter and spring, where he hosted elegant Tuesday *soirées* featuring grand receptions and sumptuous dinners. 2 3 4 5 6 7 21

## CONTRIBUTIONS TO NEUROLOGY AND NEUROPATHOLOGY

As a professor of pathological anatomy, coupled with his expertise as a neurologist, using the anatomo-clinical method, Charcot made significant contributions to the field of neuropathology. 22 23 24 He was responsible for the original neuropathological descriptions of various neurodegenerative diseases ( Table 1 ). 22 23 24 25 26 27 28 Furthermore, it should be remembered that the coronal section of the brain (“ *coupe verticale et transversale du cerveau”* ) is known as the “Charcot cut.” 23 29 30

**Table 1:** Charcot's contributions to neurology, neuropathology, and psychiatry

Subject / disease		Partners - year	Contribution
Neurology and neuropathology	Anatomo-clinical method	Vulpian, Cornil -1862	Charcot brought new vigor to the clinicopathologic tradition of the Paris school by adding to macroscopic anatomy the new dimension of histology.
	Charcot-Féré syndrome (ophthalmoplegic migraine)	Féré - 1861	Charcot, who was a migraineur, and Féré studied eight patients who presented with scintillating scotoma accompanied by changes in ocular motricity.
	Tabes dorsalis	Vulpian -1862	Charcot and Vulpian made a significant breakthrough by establishing a crucial link between specific clinical symptoms and the underlying lesions associated with *tabes dorsalis* (“ *ataxie locomotrice* ”).
	Parkinson's disease	Vulpian - 1862	Charcot corrected some of the mistakes in James Parkinson's description, such as the presence of bradykinesia and not paralysis, identified non-motor signs, and with Vulpian coined the term “ *Maladie de Parkinson* .”
	Tremor	Marie - 1862–1893	He distinguished tremor in degenerative diseases, clinical conditions, intoxication and hysteria. He was the pioneer of treatment with anticholinergics.
	Charcot-Bouchard's aneurysms	Bouchard - 1867	Charcot and Bourchard's study led to the discovery of “aneurysms that developed on intracerebral arterioles” as cause of strokes.
	Multiple sclerosis	Vulpian - 1868	Charcot provided the classic description of “ *sclérose en plaques disséminées* ”
	Amyotrophic lateral sclerosis	1869–1885	He described “ *sclérose latérale amyotrophique* ” (amyotrophic lateral sclerosis), a condition that became known as Charcot's disease
	Charcot-Joffroy syndrome	Joffroy -1873	A rare form of cervical myelopathy known as idiopathic hypertrophic cervical pachymeningitis. It has been described by Charcot and Alix Joffroy in Joffroy's doctoral thesis
	Erb-Charcot paralysis	1876	A syphilitic myelopathy with sensory and motor symptoms *(tabes dorsalis spasmodique).*
	Charcot cut	1876	The coronal section of the brain is known as the “Charcot cut.”
	Aphasia	Féré, Marie, Rummo, Bernard, Ballet - 1883	Charcot created the “bell diagram” describing the circuits of the speech process.
	Charcot-Wilbrand syndrome	1883	Charcot described a patient with posterior cerebral artery thrombosis who lost the ability to consciously reproduce images from his dreams while awake, although he could still recall dreams of words but not any imagery.
	Charcot-Marie-Tooth disease	Marie - 1886	With Pierre Marie made the initial description of the hereditary sensory-motor peripheral neuropathy. Tooth identified the pathology in the peripheral nerves three months later.
	Fugue-poriomania and epilepsies	1888	Charcot described a 37-year-old mailman with fugue-poriomania, experiencing three hour-long episodes of wandering around Paris with complete amnesia. This case is now understood as non-convulsive status epilepticus with fugue status, and it significantly contributed to the study of differentiating epilepsy from functional (psychogenic) conditions.
	Souques-Charcot geroderma	Jean-Baptist Charcot and Souques - 1891	The “ *géomorphisme cutané* ” is a variant of Hutchinson-Gilford progeria, which is characterized by loose, shiny, dry skin, subcutaneous atrophy, eunuchoid habitus, and intellectual deficiency
Psychiatry	*Idée fixes*	Gille de la Tourette - 1885	Manifestations of obsessive-compulsive disorder
	Histeria	Since 1872	He considered the disease as a result of a dynamic lesion of the brain circuits. Only patients with hysteria could be hypnotized

In 1868, Charcot provided the classic description of “ *sclérose en plaques disséminées* ” (multiple sclerosis), employing the famous anatomo-clinical method, which involves a rigorous semiological evaluation of patients followed by neuropathological studies. He collaborated with his colleague Vulpian on this outstanding description. 26 31 32 33 Similarly, Charcot masterfully described “ *sclérose latérale amyotrophique* ” (amyotrophic lateral sclerosis) between 1869 and 1885, a condition that became known as Charcot's disease in Europe. 26 34 35 36

In 1886, Charcot and his disciple Pierre Marie made the initial description of the hereditary sensory-motor peripheral neuropathy now recognized as “Charcot-Marie-Tooth disease.” They analyzed five patients and referred to the condition as “ *atrophie musculaire progressive* ” (progressive muscular atrophy). 27 28 37 Remarkably, three months later, Howard Henry Tooth (1856–1925) presented his thesis for the medical degree in England, entitled “The peroneal type of progressive muscular atrophy,” which clearly identified the pathology in the peripheral nerves. 27 28 38

Charcot played a pivotal role in defining key clinical features of the Parkinson's disease (PD), including bradykinesia and muscle rigidity, which were distinct from the muscle weakness suggested by James Parkinson (1755–1824), another polymath. Charcot's observations also encompassed descriptions of various non-motor symptoms and signs. 33 39 40 41 42 It is essential to recall that Charcot and Vulpian coined the term “ *Maladie de Parkinson* ,” known earlier as “Shaking Palsy,” in 1862. 39 43 In 1892, Charcot presented a lecture on vibration therapy, discussing his clinical experience with PD using a vibratory chair. The treatment improved sensory symptoms, sleep problems and walking ability, but had limited impact on the tremor associated with PD. Despite Charcot's efforts, the vibrating chair's use for PD was largely disregarded after his passing due to relatively few positive effects observed. 44 45 Furthermore, Charcot's investigations in the study of movement disorders included distinguishing multiple sclerosis tremor from similar manifestations seen in mercury poisoning, and hysteria. Additionally, he was the first to introduce anticholinergic treatment for tremor. 27 39 40 41 42

**Figure 3:**
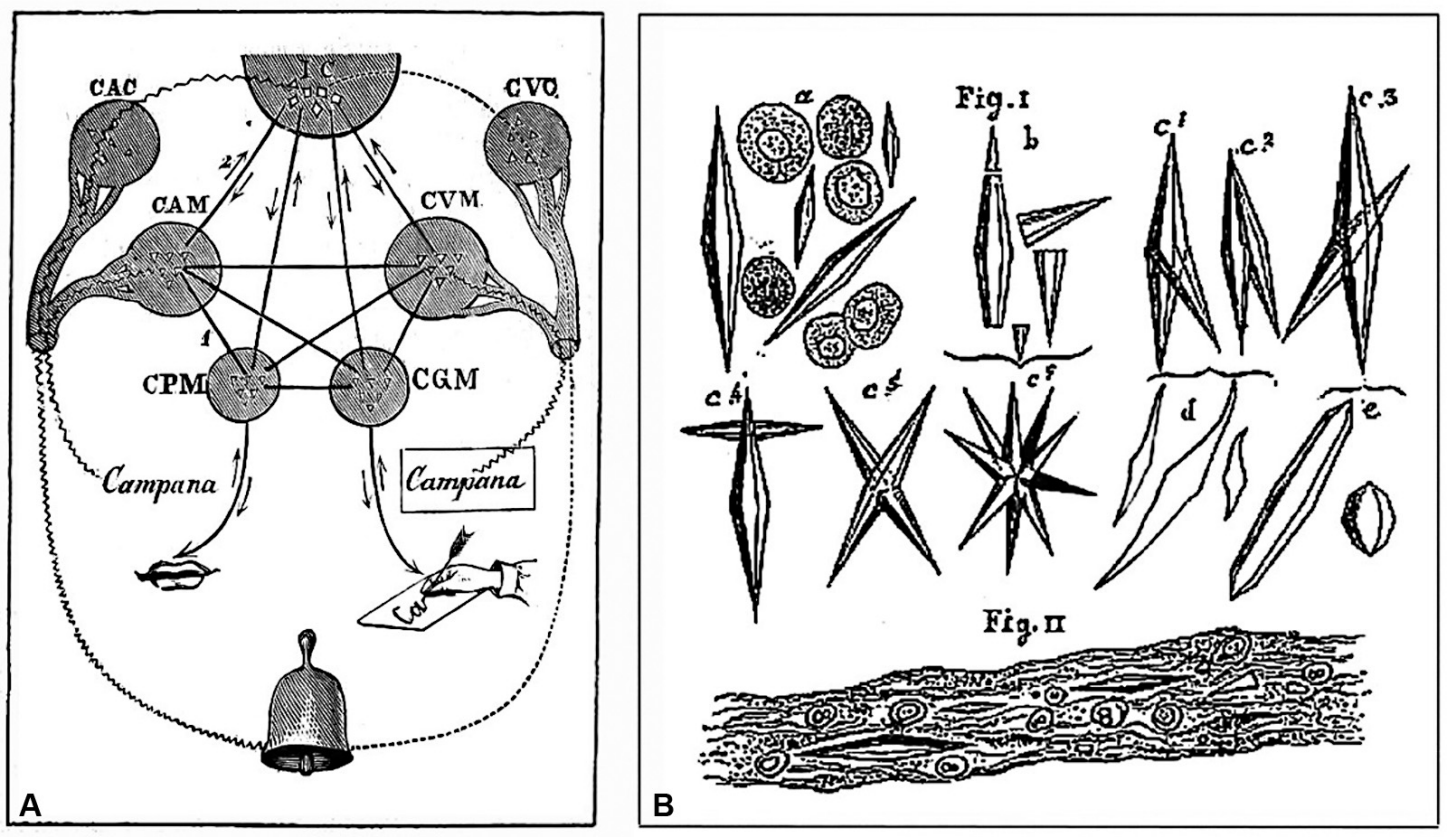
Charcot's drawings for scientific publications. ( **A** ). Charcot's Bell – Aphasias (Charcot and Rummo, 1884): The bell (campana [cloche]) rings, it is heard and seen. The centers were defined by clinicopathological analysis. Input – auditory input: CAC: center of shared hearing, and CAM: auditory center for words; visual input: CVC: center of shared vision, and CVM: visual center for words. Output – spoken output: CPM: center of articulated language; written output: CGM: center of written language. IC: ideation center. Arrows show the direction of the nervous paths connecting the centers. ( **B** ). Charcot-Leyden's Crystals (Vulpian and Charcot, 1860).

Charcot conducted extensive research on “aphasia” and delivered lectures at *La Salpêtrière* , which were later transcribed and published by Charles Féré. 46 47 48 49 50 Gaetano Rummo (1853–1917) translated and transcribed these lectures into Italian, which resulted in the book “ *Differenti forme d'aphasia* ,” published in 1884. 51 Charcot, known for his ability to make visual representations of complex concepts, received recognition as a “connectionist” or “diagram maker,” including for the remarkable “bell diagram” describing the circuits of the speech process, first presented in Rummo's book ( Figure 3A ). 51 52

In collaboration with Charles Joseph Bouchard (1837–1915), Charcot made significant contributions to the understanding of strokes, particularly cerebral hemorrhages. Their study led to the discovery of “aneurysms that developed on intracerebral arterioles.” 53 During the autopsy, Bouchard identified ruptured and intact aneurysms, providing evidence for this pathology, which was later named “Charcot-Bouchard's aneurysms.” 53 54

In addition to his extensive research on various aspects of neurology, Charcot made valuable contributions to the localization of cerebral and spinal cord diseases. 55 He conducted studies on ophthalmoplegic migraine and epilepsies. Charcot's work on ambulatory fugue-poriomania resulted in a classic description of this condition. Charcot-Wilbrand syndrome, identified by a loss of dreaming and visual agnosia, was also a subject of his research. Additionally, he studied Souques-Charcot syndrome, a variant of Hutchinson-Gilford progeria. Charcot also provided a thorough account of Charcot-Joffroy syndrome, a rare form of cervical myelopathy. Furthermore, his investigations led to the characterization of Erb-Charcot paralysis, a condition associated with spinal syphilis that manifests with sensory deficits, spastic paresis, and amyotrophy ( Table 1 ). 2 3 4 15 22 24 55 56 57 58

One of the prominent myelopathies during the 19th century was *tabes dorsalis* . 3 33 In 1862, during their early collaboration at *La Salpêtrière* , Charcot and Vulpian made a significant breakthrough by establishing a crucial link between specific clinical symptoms and the underlying lesions associated with *tabes dorsalis* (“ *ataxie locomotrice* ”). They observed degeneration or sclerosis of the posterior columns of the spinal cord and atrophy of the posterior spinal roots in affected individuals. 3 59 Around the same time, Lewis A. Sayre (1820–1900) designed a device for suspending patients for scoliosis treatment. Osip Osipovich Motschutkovsky (1845–1903) applied Sayre's method to a scoliosis patient and noticed improvements in *tabes dorsalis* . 60 Inspired by Motschutkovsky's findings, Charcot assigned Gilles de la Tourette to conduct therapeutic experiments on ataxic patients using suspension therapy. Charcot observed positive results in 14 out of 18 cases, with improvements in walking, balance, pain, and other aspects. 60 61 However, the therapy did not demonstrate objective improvement and had associated risks and fatalities. Despite the uncertainties surrounding its mechanism of action, the treatment remained in use for over a decade due to Charcot's influence before eventually losing popularity. 62

Indeed, Charcot initially believed neurological diseases were hereditary. However, the emergence of the germ theory, championed by Louis Pasteur (1822–1895), in the late 19th century, brought a paradigm shift to French medicine, suggesting microorganisms as disease agents. Charcot was skeptical but not entirely opposed to the new theory, staunchly defending “hereditarianism” for years. 62 In 1882, Jean Alfred Fournier's (1832–1914) association of *tabes dorsalis* with syphilis marked a turning point, supporting an infectious etiology, contributing to the growing acceptance of the germ theory, including those within Charcot's circle at *La Salpêtrière* , such as Bouchard and Pierre Marie. 62 63 As a result, Charcot's adherence to hereditarianism and resistance to the new theory led to a certain decline in his political power and influence within French academic circles. 62 64

## CONTRIBUTIONS TO NEUROPSYCHOLOGY AND PSYCHIATRY

Charcot's contributions in this area of neuropsychology and psychiatry are numerous, as example, descriptions of the so-called “fixed ideas” (“ *idée fixes”* ), manifestations of obsessive-compulsive disorder, observed in patients with tics and Tourette's syndrome ( Table 1 ). 2 3 4 15 24 65 66

Charcot's significant contributions to the study of “hysteria” are evident in his research on this clinical condition, now recognized as functional disorders, a term originally coined by Charcot as “neurosis.” 4 15 24 66 67 68 69 Charcot refuted the muscular origin proposed by Pierre Briquet (1796–1881) and disagreed with the traditional “uterine” explanation. Instead, he aligned with the concept of “ovarian hyperaesthesia” suggested by Charles Négrier (1792–1862). 70 71 72 Charcot advocated vigorous compression of the ileo-hypogastric region for the treatment of hysteria and differential diagnosis with epileptic seizures. 70 71 72 73 Nonetheless, neither Charcot nor his disciples formulated any hypotheses explaining the underlying mechanism by which abdominal compression influenced their patients. 70 In the later stages of his life, Charcot underwent a paradigm shift in his understanding of hysteria, conceptualizing the condition as a consequence of dynamic brain circuit lesions. 4 15 24 66 67 68 69

Influenced by Charles Richet's (1850–1935, future Nobel Prize winner in 1913) research on “provoked somnambulism,” Charcot began using hypnosis as a therapeutic tool for hysterics in 1878. 74 75 However, his convictions were not always well accepted by another important research group in this area, the *École de Nancy* , led by Hippolyte Bernheim (1837–1919). Debates surrounding hypnotism between Charcot's school and the *École de Nancy* garnered significant public attention, and a well-known crime added to the sensationalistic nature of the discourse. Charcot maintained a viewpoint opposing Bernheim's, asserting that only patients with hysteria could be successfully hypnotized. 74 76 Regrettably, Charcot's approach to discussing hypnosis was combined with pseudoscientific subjects such as clairvoyance and spiritism during his lectures, which drew disapproval from the academic community. 77 78 His research on hysteria and hypnosis at *La Salpêtrière* influenced Gilles de la Tourette and others, while some of his close disciples, including Féré, Pierre Janet (1859–1947), Alfred Binet (1857–1911), and later Babiński, distanced themselves from the Nancy-Paris controversy. 76 78 79

## CHARCOT SEMIOLOGIST

Throughout his career, Charcot presented his cases with rich detail on semiology. He not only described new signs, but also reinterpreted previously described findings. 2 3 6 7 Charcot described what is known as the biliary triad or “Charcot's triad.” 80 The triad is characterized by the presence of recurring abdominal pain in the upper right quadrant associated with fluctuating jaundice and intermittent fever with shivers, and it is associated with the presence of acute cholangitis caused by choledocholithiasis. 80 In patients with *sclérose en plaques* , the presence of nystagmus, intentional tremor, and dysarthria ( *staccato* speech) has also become known as “Charcot's triad.” The “Charcot's sign” occurs when there is eyebrow elevation in peripheral facial paralysis. 8 In 1890, Charcot described areas of the body whose compression causes hysteria, the “Charcot's zones.” These areas overlapping fibromyalgia tender points ( Table 2 ). 81

**Table 2:** Charcot's semiology: signals symptoms and maneuvers

Name	Year	Description
Charcot-Marie's signal	1883	Fine, rapid generalized tremor in Basedow-Graves' disease
Charcot's vertigo	1876	cough-induced dizziness, laryngeal spasm
Charcot's signal	?	Eyebrow elevation in peripheral facial paralysis
Charcot's triad I	1868	Nystagmus, intentional tremor and dysarthria (“chanted speech”) in multiple sclerosis patients.
Charcot's triad II	1877	Biliary triad. Presence of recurring abdominal pain in the upper right quadrant associated with fluctuating jaundice and intermittent fever with shivers, and it is associated with the presence of acute cholangitis.
Charcot's zones	1890	Areas of the body whose compression causes hysteria (Charcot's ovarian point)
Clonus	1862	“Epileptoid trepidation of the foot”
Ear tophaceous	1860	Tophaceous deposits in the ear lobes (gout).
Charcot's edema	1892	Local and very painful edema with a bluish appearance of the extremities, seen in hysterical paralysis.
Charcot's intermittent hepatic fever	1877	Continuation of Charcot's triad II

A Charcot's innovation for semiology was the systematic taking of residents' temperatures with a mercury thermometer, rather than simply by hand. His intern for 1868, Bourneville, would make this the subject of his thesis and other subsequent publications. 8 The ocular fundus examination, with an ophthalmoscope, was a new technique invented by Hermann von Helmholtz (1821–1894), in 1851. The importance of fundoscopic examination to the clinical practice of neurology was appreciated by Charcot, who was an early adopter of the ophthalmoscope, and found it useful in some trying differential diagnosis, as in the lecture “ *De l'amaurose tabétique”* , where he wrote “However, ophthalmoscopy, in this situation, came to bring us a decisive contribution.” 82 83

## CONTRIBUTIONS TO INTERNAL MEDICINE

Charcot described many diseases in different areas of internal medicine, causing him to be defined as the “discoverer of diseases” ( Table 3 ). 15

**Table 3:** Charcot's contributions to internal medicine

Subject / disease		Partners - Year	Contribution
**Rheumatology**	Gout	Piorry - 1853	Charcot distinguished gout from chronic rheumatism (rheumatoid arthritis)
		Garrod- 1867	Gout in patients with lead poisoning
	Charcot's joint	1881	Arthropathy in patients with *tabes dorsalis* (neurosyphilis)
	Charcot's arthopathy	1868	Progressive degenerative arthropathy associated with various types of neuropathic diseases, more commonly with diabetes mellitus. “Diabetic Charcot's foot.”
**Endocrinology**	Basedow-Graves' disease	1856	He argued that the cause of the disease was not in the heart, but in the thyroid
		Hirsch - 1859	Unlike Trousseau, who coined the eponym Graves' disease, Charcot advocated the eponym Basedow's disease.
	Addison's disease	Vulpian - 1857	Microscopic findings in the adrenal glands.
**Nephrology**	Chronic renal insufficiency	1858	Retinal damage and thrombosis of the central retinal artery
**Hematology**	Melanemia	1857	Charcot recognized this blood change as present in several diseases, such as malaria. It could originate in the spleen, or liver, or kidneys.
	Charcot-Leyden crystals	Robin -1853 Vulpian -1860	Crystals found abundantly in patients with acute leukemia. Later found by Leyden in asthmatic patients.
**Angiology**	Pulmonary embolism	Ball - 1858	In a pulmonary embolism case, Charcot described the pathophysiology showed that the clot obstructing the artery was from venous phlebitis of a lower limb. He recognized the elevation of fibrin as a factor promoting clot formation.
	Intermittent arterial claudication	1859	Charcot elucidate the pathophysiological mechanism of ischemia.
	Charcot-Weiss-Baker syndrome	1872	He described a type of syncope triggered by coughing as well as syncope triggered by compression of the carotid sinus.
**Infectiology**	Parasitosis	Davaine - 1852	They presented cases of hydatidosis and echinococcosis.
	Gangrenous dissecting pneumonia	Ball - 1860	They envisioned septic emboli before the era of microbiology.
	Typhoid fever	Vulpian - 1862	Description of valvular damage, particularly tricuspid.

**Figure 4:**
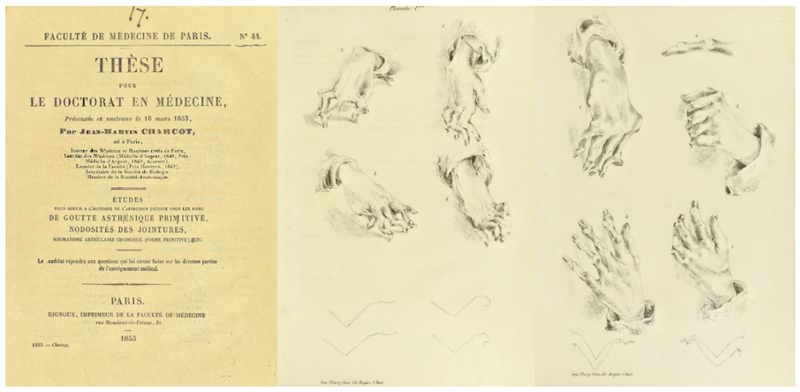
Charcot's thesis, defended at the Faculty of Medicine at the University of Paris in 1853. Charcot drew the joint deformities to illustrate his doctoral thesis. (Charcot, 1853).

His doctorate thesis, defended at the University of Paris in 1853, focused on the “ *Goutte Asthénique Primitive, Nodosités de Jointures, Rhumatisme Articulaire Chronique”* , providing a classical description of progressive chronic rheumatism and differentiating it from cases of gout, under the chairmanship of Piorry. 2 3 4 8 84 His talents as a draftsman are revealed in the plate of hands with deformed fingers illustrating his thesis ( Figure 4 ). 8 Throughout his career, Charcot remained knowledgeable about rheumatic diseases and gout, and in 1863, he reported Alfred Baring Garrod's (1814–1917) observation of the high frequency of gout in those with previous signs of lead intoxication. 8 85 Additionally, Charcot described an arthropathy in patients with *tabes dorsalis* , known worldwide as “Charcot's joint,” and another articular disease related to diabetic foot arthropathy, referred to as “Charcot's arthropathy” or “diabetic Charcot's foot,” which is associated with various types of neuropathic diseases, but which is undoubtedly much more common in patients with diabetes mellitus. 2 3 4 20 84 This condition predominantly affects the feet, leading to structural deformities and the risk of ulcers and even osteomyelitis. 2 3 4 84 86 87 88

In 1835, Robert James Graves (1796–1853) described cases of tachycardia and thyroid enlargement in young women, attributing them to a cardiac lesion and creating the cardio-vascular theory with William Stokes (1804–1878). 89 In 1840, Carl A. von Basedow (1799–1854) associated palpitation of the heart, exophthalmos, and goitre, known as the Merseburg Triad, and suggested a cause related to a “dyscrasia of the blood.” 90 In 1856, Charcot presented a case series of this disease, rejecting the cardiac cause and focusing on structural changes in the thyroid arteries. He proposed increased gland activity due to vasomotor nerve stimulation. In 1859, he reported a case with a fatal meningeal hemorrhage completing the disease's evolution. Charcot also described different tremors in the disease, leading to the term “Charcot-Marie's signal.” 8 Armand Trousseau (1801–1867) admired Graves and Stokes, using the term “Graves' disease” in his clinical lectures. 8 When Graves' textbook was translated into French, Trousseau wrote the foreword in which he commended to readers the work of Graves and indeed first used the term “Graves' disease.” 89 In contrast, Charcot followed the Germanic school and endorsed August Hirsch's (1817–1894) proposal to name the condition “Basedow's disease.” This eponym is still used in non-Anglophone countries to this day. 8

With Vulpian, Charcot presented an observation of Addison's disease to the *Société de Biologie* in 1857. Macroscopically, the adrenal glands appeared normal, but microscopic observation showed degeneration: “after the ordinary symptoms of Addison's disease, the adrenal capsules would be given as healthy, without having been examined under the microscope or treated with suitable reagents. From the point of view of theory, this should be regarded as untrue.” 8

The use of the microscope, for which Charcot always had great enthusiasm, also contributed to his important discoveries. In 1857, he published an article on “ *melanemia* ” where he identified colored corpuscles derived from the regressive metamorphosis of red blood cells in certain pathological conditions. He attributed melanemia to the spleen and liver's activity and associated it with nephritis in malarial fever cases. However, Charcot rejected melanemia as the cause of neuropsychic disorders during malaria and wondered whether it should be considered a symptom or a disease, mainly focusing on its relation to fevers. 8

In 1853, Charcot and Charles Philippe Robin (1821–1885) presented a case of acute leukemia to the *Société de Biologie* . During the autopsy, Robin noted that in the blood of the right ventricle, there was a large quantity of blood crystals, which were very regular in shape and slightly colored yellowish-red. These crystals were also found abundantly in the spleen tissue, forming considerable clusters visible only under the microscope. In 1860, Charcot and Vulpian, referring to another leukemia case, described these crystals as “very elongated octahedrons, very regular in shape and fairly uniform,” measuring 6 to 8 μm ( Figure 3B ). 91 These crystals later became known as Charcot-Leyden crystals in honor of Charcot and Robin's 1853 description, with the addition of the name of Ernst Victor von Leyden (1832–1910), who identified them in the sputum of asthmatics in 1872. 92 Similarly, Charcot-Neumann crystals, which contain phosphate crystals, were described in semen. 4

Intermittent claudication of arterial origin (“ *claudication intermitente par oblitération artérielle”* ), originally described by Charcot (“Charcot's intermittent claudication *”* ), is characterized by the presence of pain, discomfort, weakness, or leg cramps, mainly after physical exercise or walks and disappear after rest. 84 93 94 Charcot described this disease in detail in a soldier who had been injured by a firearm and who developed an aneurysm that formed a thrombus blocking the iliac arteries. 84 93 94 95 96 He based his study on the description, published in an article in 1831 by the Parisian veterinary surgeon, Jean-François Bouley, of claudication in a horse that pulled a carriage through the streets of Paris and had difficulty using its hind feet whenever it had to exercise more strenuously. The animal presented thrombi obstructing the femoral arteries. 95

François-Amilcar Aran (1817–1861) wrote his dissertation in 1853 on the causes of sudden death, including pulmonary embolism, but without specifying its pathophysiology. Benjamin Ball (1833–1893) and Charcot showed in 1858 that the origin of the clot obstructing the pulmonary artery was venous phlebitis of a lower limb. Charcot recognized the elevation of fibrin as a factor favoring clot formation, even though the physiology of hemostasis was still in limbo. He noted the higher frequency of these diseases in young people, especially with a traumatic injury. 8

Another contribution related to the field is Charcot's edema, a painful and bluish edema described in women with hysterical paralysis. 84 93 Charcot gave a detailed description of vasovagal syncope, which is characterized by transitory attacks of significantly reduced heartbeat, reduced arterial pressure, and loss of consciousness. 84 Then, in 1872, he helped clarify a syncope triggered by coughing, by compression of the carotid sinus (carotid sinus hypersensitivity syndrome), described by Johann Nepomuk Czermak (1828–1873) in 1866. Its pathophysiology was comprehensively described by Soma Weiss (1898–1942) and James Porter Baker (1902–1988). 97 This syndrome is also known as the Charcot-Weiss-Baker syndrome. 84 88 Furthermore, Charcot conducted investigations on sore pressure. 98

Charcot made significant contributions to various areas of internal medicine, including nephrology and infectology ( Table 3 ). In his early lessons, he discussed fever, conditions like la *gravelle biliaire,* pneumonia, and more, without the knowledge of microbiology, which had not yet been established. Charcot and Ball presented a case in 1860 of a woman with heart failure and mitral stenosis who underwent puncture for ascites. Unfortunately, the procedure led to complications, including erysipelas and gangrenous necrotic tissue, resulting in her death. They used the term “gangrenous dissecting pneumonia,” introduced by the German Hermann Lebret (1813–1878) in 1845, envisioning septic emboli before the era of microbiology, suggesting the spread of septic liquids from a primitive focus or blood clots carrying gangrenous fluid. 8

He can also be considered to have pioneered the study of diseases of the elderly, although his contributions in the fields of geriatrics and gerontology are scarce, focusing mainly on gout and chronic rheumatism. 31 99 100 He was also one of the pioneers in establishing rehabilitation clinics with physiotherapy, speech therapy, hydrotherapy, and electrical stimulation of paralyzed muscles. 3

## CHARCOT: AN INTELLECTUAL

In his book published in 1988, Paul Johnson, defines intellectuals, presents a series of them, and highlights their moral qualifications, capacity for discernment, and characteristics that qualify them to guide humanity. 101 Charcot had all the basic characteristics for be defined as an intellectual. In parallel with his scientific activities, Charcot had a great appreciation for the arts in general and vast literary and artistic aptitude, and he was also fluent in five languages. 2 3 4 5 6 7 102 103 104 105

He was a man of strong personality and his features emanated a respectable figure. Charcot may be defined as austere, reserved, introvert, taciturn, shy, authoritarian, associated with a competitive temperament, permeated by a skepticism in relation to the treatment of many neurological diseases, and sometimes, with expressions of irony and even sarcasm, in the relationship with other neurologists, and, paradoxically, an intense love for animals. 2 3 105 106 107 108 109 110

Charcot's characteristics regarding the arts, in particular painting and sculpture, were quite eclectic. 2 3 6 7 He had great admiration for the sculpture of ancient Greece, Italian paintings of the Renaissance period, as well as Belgian and Dutch paintings. 2 One of his favorite painters was Delacroix. 2 Although Charcot lacked the ability to play musical instruments, he had an exceptional taste for music. His favorite classical composers were Mozart, Beethoven, Rameau, Gluck, as well as César Franck and Hector Beriloz. 2 3 6 7 In the area of literature, he knew all of Shakespeare's works, of which he was a great admirer and claimed him as his favorite writer. Charcot had a great interest in the area of philosophy, and liked the classic Greek and Latin books, particularly by Plato and Seneca. 2 3 6 7

Even though he was not politically engaged, Charcot had liberal ideas, and even though he was tolerant in religious matters, he was clearly anti-clerical. 3 106 111 Nevertheless, his wake in the chapel of the *La Salpêtrière* and burial in the *Montmartre* cemetery were performed within Catholic norms. 2 3 6 7 111 112 Some biography data suggest that Charcot, in the final phase of his life, came to admire Buddhism. 113

## CHARCOT: THE ARTIST

Charcot's inherent artistic abilities were a result of his exceptional visual perception and prodigious memory, as documented in various works throughout his career. 2 3 6 7 102 103 104 105 Notably, one of his disciples, Henry Meige (1866–1940), published a book in 1925 titled “ *Charcot Artist* ,” wherein he showcased a collection of original drawings by Charcot. These drawings encompassed environments, landscapes, family members, colleagues, church sculptures, and several caricatures, some of which exhibited a sense of self-ridicule. 104 The book “ *Charcot - Une vie avec l'image* ,” published by Catherine Bouchara in 2013, provided substantial insight and a comprehensive perspective. It also presented numerous drawings by Charcot, showcasing his astute observation of patients' postures and neurological signs, always correlated using the renowned anatomo-clinical method. The book featured various drawings of patients with hysteria, family members, in particular his son, Jean-Baptiste and daughter, Jeanna, landscapes from his international travels, and several caricatures. 105

Charcot's own book titled “ *Huit jours au Maroc* ” (“Charcot in Morocco”) chronicled his trip to Morocco in 1887 and contained numerous drawings, featuring landscapes and local characters, notably from the Jewish community. 102 114 Furthermore, Charcot authored an article later compiled into a book titled “ *La foi qui guérit* ” (“Faith Healing”). 115 Contrary to his prior skepticism regarding neurological disease treatments, Charcot objectively explored the significance of faith as an auxiliary element in treating patients with neurological ailments. He included conversion pictures related to hysteria, which foreshadowed the later development of psychosomatic medicine. 115

Charcot collaborated with his assistant Paul Richer on the book “ *Les Démoniaques dans l'Art* ,” published in Paris in 1887. 103 This work, illustrated by Richier, depicted hysterical symptoms in religious and religious art, including Charcot's descriptions of hysteria in religious contexts and his “hysterical saints.” It also explored Charcot's contributions to the field of functional disorders and their implications. 103 116 Another joint publication by Charcot and Paul Richer in 1889 was “ *Les Difformes et le Malades dans l'Art* ” (“The deformed and diseases in art”), further discussing the intersection of science and art. 117

Moreover, Charcot actively encouraged his disciples, such as Bourneville and Paul-Marie-Léon Regnard (1850–1927), to share the neurological findings of their group at *La Salpêtrière* through various journals. The “ *Iconographie Photographique de la Salpêtrière* ,” created in 1876, particularly emphasized hysteria, and later, the “ *Nouvelle Iconographie de la Salpêtrière* ” covered a wider range of neurological diseases. 3

### Charcot Caricaturist

**Figure 5:**
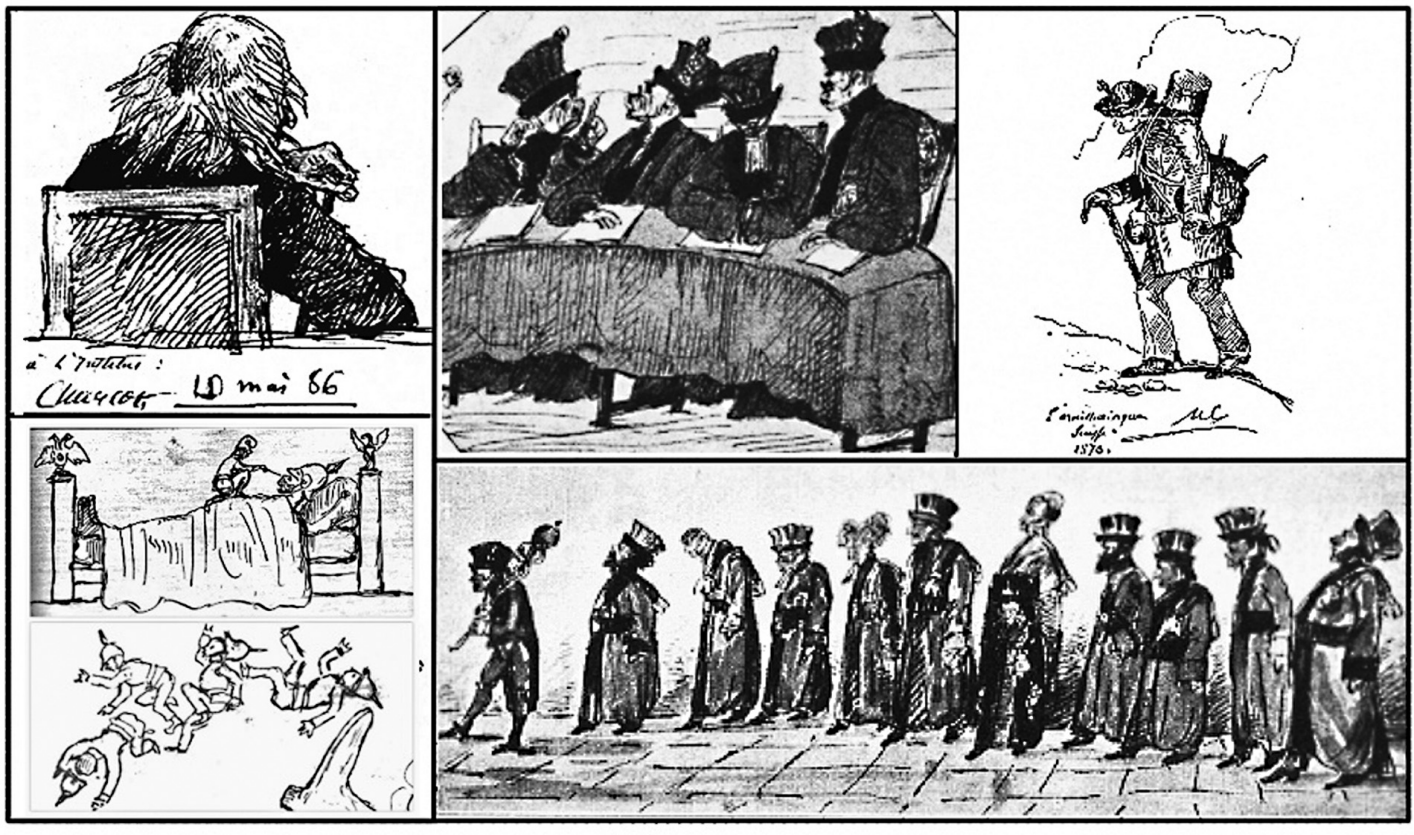
Caricatures by Charcot. Clockwise from top: (1) Professor Michel Eugene Chevreul (1786–1889) during Charcot's visit to a Tuesday session at the Academy of Sciences in Paris around 1885; (2) “The Areopagus,” Charcot drew his medical school colleagues as apes; (3) An old companion from his youth nicknamed “Platypus,” on an excursion in the mountains of Switzerland; (4) Faculty procession: his colleagues at the Paris medical school in pompous costumes during a procession; (5) The final two images portray his indignation during the Franco-Prussian war (1870): the first is untitled, depicting a tiny French soldier atop an enormous, inert Prussian one, and the second he called “ *L'Avenir* ” (“The Future”). Source: Walusinski, personal collection.

Charcot's relationship with various aspects of art in general, which at first started as a hobby and later became more scientific, also turned toward caricature. 102 104 105 In this area, his works demonstrate significant artistic skills, which are clearly evident in Henry Meige's book entitled “ *Charcot Artiste”* and the more recent text by Catherine Bouchara entitled “ *Charcot - Une vie avec l'image”* . 104 105 In 2021, Teive et al. 102 published a historical note to discuss the artistic side of Charcot through his caricatures. This historical note briefly describes eight caricatures during the second half of the nineteenth century in Paris, demonstrating his artistic gifts blended with humor, satire, irony and sarcasm. 102 Figure 5 fully demonstrate these characteristics.

## CHARCOT: THE LOVER AND PROTECTOR OF ANIMALS

Charcot was known for his strong sensitivity toward animals and was against hunting and bullfighting due to the unnecessary cruelty inflicted on animals. Charcot's compassionate nature extended to his love for animals, and he treated them with tenderness and care. 6 7 108 109 Guinon 6 reported the scene of Charcot being seen threateningly running behind a duck to catch a frog trapped in its beak.

In his personal life, Charcot had two dogs and a small female monkey named Rosalie, given to him as a gift by Pedro II, the Emperor of Brazil. He developed a close bond with Rosalie, and she would join him at the table during his meals. Charcot would ensure she had enough food and delighted in her playful antics, finding joy when she grabbed nuts or bananas from his plate. The presence of animals, particularly dogs, was a constant in Charcot's home at 217 Boulevard Saint-Germain in Paris. 2 6 7 109 110

Charcot's approach to scientific research was distinct from his contemporaries. He firmly opposed vivisection and experiments involving animals at *La Salpêtrière* , where most of his neuroanatomical and neuropathological studies were based on human autopsies. 3 6 7 109 110 His stance against animal experimentation was evident in his office, where a sign painted by his wife clearly stated, “You will find no dog laboratory here.” 3 6 7 108 109 This sign symbolized his strong conviction against the use of animals for experimental purposes. Despite his dedication to human autopsies and his contributions to the field of neurology, Charcot's opposition to vivisection received criticism from some members of the scientific community, especially those associated with the *École de Médecine* . 62

## DEATH

Two months following his tiring and counterintuitive journey to England, his wife asked him to rest, as his angina attacks were becoming more frequent. Charcot embarked on a cultural expedition with two of his pupils Isidore Straus (1845–1896) and Maurice Debove (1845–1920) to Vezelay and its basilica, in Burgundy. Tragically, on August 16th, 1893, at three o'clock in the morning, he experienced a severe pulmonary edema as a consequence of cardiac insufficiency secondary to myocardial necrosis. He passed away in a modest guesthouse located by the lake des Settons. Among his co-morbidities were obesity, heavy smoking, and lombalgia. 2 3 With his demise, the world lost the esteemed “ *César de la Faculté* ,” the renowned “ *Napoleon des névroses* ,” and the venerable “father of Neurology.” 3 62 118

In conclusion, the celebration of the 200th anniversary of the birth of Jean-Martin Charcot is approaching, and it is scheduled to take place with great dignity in Paris in 2025 during the annual meeting of the International Society for the History of the Neurosciences ( www.charcot2025.fr ). It is important to remember his numerous contributions to internal medicine, neurology, neuropathology, neuropsychology and neuropsychiatry. These accomplishments, in addition to his recognized and expressive artistic production and socio-cultural influence, certainly deserve him the acknowledgment as a polymath.
